# A journey of partnership: Supporting Indigenous science in Western, colonial-grounded academic institutions

**DOI:** 10.1371/journal.pone.0334949

**Published:** 2025-10-22

**Authors:** Heather J. A. Foulds, Leah J. Ferguson, Colin P. T. Baillie, Alexandra King, Lucie Lévesque, Tricia McGuire-Adams, Tara-Leigh F. McHugh, Kate Storey, Treena Delormier

**Affiliations:** 1 College of Kinesiology, University of Saskatchewan, Saskatoon, Saskatchewan, Canada,; 2 School of Kinesiology and Health Studies, Faculty of Arts and Science, Queen’s University, Kingston, Ontario, Canada,; 3 College of Medicine, University of Saskatchewan, Saskatoon, Saskatchewan, Canada,; 4 Faculty of Kinesiology & Physical Education, University of Toronto, Toronto, Ontario, Canada,; 5 Faculty of Kinesiology, University of Calgary, Calgary, Alberta, Canada,; 6 School of Public Health, University of Alberta, Edmonton, Alberta, Canada,; 7 School of Human Nutrition, McGill University, Montréal, Quebec, Canada; Public Library of Science, UNITED STATES OF AMERICA

## Abstract

**Introduction:**

Engagement of Indigenous science (Indigenous research, knowledges, and processes) is increasingly recognized within institutions of higher learning, funding bodies, and publication outlets. Respectful and authentic support for Indigenous science requires transformations of Western, colonial-grounded knowledge and knowledge processes, bodies, and institutions to meaningfully and appropriately include Indigenous ways of knowing, being, and doing.

**Objective:**

The objective of this study was to identify fundamental changes required to support Indigenous science within Western, colonial-grounded academic institutions focusing on “Identity and Colonial Institutions”.

**Methods:**

In 2019, a three-day gathering of 18 Indigenous and non-Indigenous researchers and trainees, Elder/knowledge helper/knowledge keepers, and community members was held in Treaty 1 territory and birthplace of the Métis Nation. Through talking circles, participants shared their experiences working with Indigenous communities on projects involving Indigenous knowledges.

**Results:**

Thematic analysis drew meaning from the talking circles, identifying four main themes: 1) Building Bridges; 2) Institutional Practice; 3) Original Knowledges; and 4) Multifaceted Identity. Focusing on “Identity and Colonial Institutions” stemming from these themes, recommendations for supporting Indigenous science were identified around four central actions: 1) Embedding respectful and authentic support; 2) Acceptance, endorsement, incorporation, and education among the broader research community; 3) Prioritizing and valuing Indigenous research, knowledges, processes, and contributions; and 4) Privileging of multiple worldviews.

**Conclusions:**

Institutions, funding agencies, journals, and all individuals, organizations, and entities involved in research are encouraged to enact these recommendations and take action to support Indigenous science.

## Introduction

Indigenous research, knowledge systems, and processes, known as Indigenous science, is a body of knowledge that has developed and evolved since time immemorial through direct interactions with the natural world, generally rational, methodical, and empirical, advancing the respective Indigenous society and culture of whom the Indigenous science belongs [1,2]. Indigenous science is sometimes referred to as Ethno science, traditional knowledge, or Indigenous Knowledge, among many other terms [3]. Within research institutions and programs on Turtle Island (lands now known as Canada, the United States, and Greenland), traditional territories and homelands of First Nations, Inuit, Métis, American Indian and Alaskan Native Peoples, consisting of many distinct and diverse nations [4]. Indigenous science (Indigenous research, knowledges, and processes) is a growing area of study, engaging and exploring Indigenous knowledge systems, ontologies, epistemologies, and methodologies [5,6].

Traditionally, Western knowledge systems in academia have been grounded in colonial processes, ideals, and research metrics and protocols [7]. Enfranchisement laws from 1876–1961 legislated that First Nations Peoples legally recognized by the Government of Canada would lose their “Indian status” if they attended universities [8,9]. Further, modern colonial science has attempted to separate nature and society, positioning modern science and affiliated knowledge systems taught and used within research institutions, as separate from the nature around us and culture we are engaged in [10]. Pervasive undercurrents and beliefs that modern colonial science is superior to other knowledge systems and forms of science further entrench research institutional approaches that privilege only modern colonial science [11]. Subsequently, the incorporation and inclusion of Indigenous science in academia has been missing until recently [5,6].

Although Indigenous science has recently been recognized in academia and academic research, Western axiologies, ontologies, epistemologies, and methodologies are pervasive, unconsciously, and uncritically accepted. Resistance to structural change incorporating Indigenous science into Western, colonial research institutions is deeply embedded within decision making structures, funding models, academic rewards systems, and other systemic structures [12,13]. Incorporating Indigenous science into Western, colonial research institutions requires fundamental paradigm shifts, toppling hegemony of modern colonial science [1]. Transforming Western, colonial-grounded research paradigms to include, respect, and privilege Indigenous science brings more wholistic, interdisciplinary, and transdisciplinary approaches to research inquiry [5,6]. Indigenous research paradigms offer safe and supportive spaces for researchers, and particularly community-engaged researchers, to prioritise Indigenous community knowledge; emphasize the integral roles of relationships; and address the inequities, marginalization, and social, political, economic, and planetary injustices faced by Indigenous communities [14,15].

Despite signaling recognition of the value and importance of Indigenous science, knowledge systems, and worldviews through Indigenous-targeted funding initiatives [16], research institutions continue to support and evaluate Indigenous science through Western, colonial-grounded research standards and needs. Privileging Western standards, structures, and supports without consideration for Indigenous knowledge systems position Indigenous research as lower quality, undermining and devaluing Indigenous knowledge systems, and place undue burdens, challenges, and barriers on Indigenous researchers and researchers working in areas of Indigenous research [15,17].

Many universities have embarked on “Indigenization” strategies, transformative movements to expand narrow conceptions of knowledge to include Indigenous perspectives, knowledges, and ways of being [18], to make universities more accessible to and inclusive of Indigenous Peoples, science, and worldviews [8]. Indigenous scholars, however, continue to face challenges in attaining tenure (21% of Indigenous faculty with tenure vs. 37% of non-Indigenous faculty), and take longer to attain tenure and progress to full professor than non-Indigenous scholars [19,20]. Further, despite often achieving more publications and tri-council funding than Western-focused researchers, Indigenous researchers and researchers engaging in Indigenous science are often undervalued [20]. Many Indigenization initiatives are structured as superficial top-down approaches, with significant limitations as to how and where Indigenous knowledges, science, and worldviews are incorporated within Western, colonial-grounded academic institutions [21–23].

Engaging in supportive, respectful, and authentic shifts within Western, colonial-grounded academic institutions is required to fully support Indigenous science in these arenas. Such shifts must reflect genuine deeply engrained structural changes to inherently incorporate Indigenous science in ways that will be lasting, foundational to research institutions, and will accurately represent and enact Indigenous science. Multiple approaches to Indigenization within academic institutions exist – from inclusion: increasing the numbers of Indigenous Peoples hired, to reconciliation: attempting to find common ground between Indigenous and Western, colonial-grounded ideals, to decolonial Indigenization: complete overhauls of current practices [24]. Meaningfully incorporating Indigenous science will require decolonial Indigenization, transforming institutions and reorienting knowledge systems. The objective of this study was to identify fundamental changes required to support flourishing of Indigenous science within Western, colonial-grounded academic institutions specifically from perspectives of Identity and Colonial Institutions. The actions and recommendations identified through this study serve as potential starting points for such decolonial Indigenization.

## Materials and methods

This research was conducted under the guidance and direction of a Cree Elder, and a Kanien’kehà:ka (Mohawk Nation citizen) knowledge helper, and facilitated by a Kanien’kehà:ka knowledge keeper. While Elder and Knowledge Keeper are current academic terms describing Indigenous individuals who hold, practice, transmit, and support Indigenous knowledges, teachings, cultural practices, an respected positions reflective of their knowledge and expertise within their specific Indigenous communities, varied terms are preferred across communities, such as Old Ones, Wise Ones, Holy Ones, or knowledge holders, and terms used should reflect specific individual and community practices, cultures, and self-identification, consistent with the multiple distinct titles used in this study. This study was part of a larger project evaluating Indigenous science in academia, as part of the Social Sciences and Humanities Research Council (SSHRC) Special Call for Indigenous Research Capacity and Reconciliation – Connection Grants, used to develop a report to the Canada Research Coordinating Committee (CRCC). The CRCC, a federal body, provides a senior strategic forum to advance federal research priorities and coordinate policies and programs of Canada’s research funding agencies and the Canada Foundation for Innovation [25]. Ethics approval was obtained from the General Ethics Board at Queen’s University (GSKHS-273–17).

On January 4–6, 2019, a community of practice was gathered in Treaty 1 Anishinaabeg and Nehiyaw territory and birthplace of the Métis Nation to discuss emerging issues related to the rigorous use of Indigenous knowledge systems and methodologies within Indigenous research. This community-grounded research was conducted by, with, and about Indigenous researchers and allies, including Elder/knowledge helper/knowledge keepers, an artist, community members, researchers, and trainees from diverse Indigenous Nations (Kanien’kehà:ka, Cree, Métis, Anishinaabe, Algonquin, and Mi’kmaq Nations) and geographies (with personal and family ties to lands from Pacific to Atlantic coasts, including seven colonial provinces). Consistent with current academic practice, the present study uses the term ally to describe non-Indigenous individuals engaging in and supporting Indigenous Peoples, rights, practices, and science; however, within and across Indigenous and non-Indigenous communities and Peoples, different terms are preferred such as supporters, accomplices, advocates, or settlers, and terms used should reflect specific Indigenous community’s and non-Indigenous individual’s practices, cultures, and self-identification. Participants were diverse across genders, ages, and research programs, including funding across national funding programs (SSHRC, Canadian Institutes for Health Research (CIHR), Natural Sciences and Engineering Research Council (NSERC)), and involved in designing and conducting Indigenous science at public universities.

Prior to the gathering, four research team members (one man, three women; one First Nations, three allies) unable to attend the gathering connected remotely to develop the structure and questions posed at the gathering. Gathering invitees were emailed the preliminary questions guiding talking circles in advance. The gathering began with an unguided walk to the Museum of Human Rights, enabling connection with lands, relations, spirit world, and with fellow participants; gathering attendees engaged in land-based activities throughout the gathering.

### Participants

Of 22 invited participants, 18 attended the gathering, all providing oral consent followed by written informed consent prior to participation and were provided a gift of tobacco as a cultural form of consent. Participants, 5 men and 13 women, included 11 First Nations, 2 Métis, and 5 allies. The gathering began with participant introductions and self-descriptions, participants developed rapport and shared their background and experiences related to Indigenous science. Led by a Cree Elder, a discussion engaging all participants together was held to develop shared understanding of “What are Indigenous knowledges”.

### Data generation

Representing this project’s collaborative partnership of Indigenous Peoples across Turtle Island, shared leadership of a Cree Elder and Kanien’kehà:ka knowledge helper and knowledge keeper, incorporated aspects of Cree and Kanien’kehà:ka cultures yielding a combined approach to data generation. Talking circles guided by Haudenosaunee decision-making processes were employed among three groups of six participants, each including a facilitator, note-taker, and reporter, and at least one Indigenous faculty researcher and one trainee. Talking circle practices are similar to sharing circles, healing circles, and peace-making circles across Indigenous Nations, reflecting diverse spiritual and cultural protocols [26]. This study used talking circles, rather than sharing circles to reflecting the sacred ness of Nehiyaw (Cree) sharing circles where conversations cannot be taken outside of the sharing circle thus preventing research applications of such conversations. While multiple rounds of talking circles were conducted for the larger project, the current study focuses on talking circles exploring “Identity and Colonial Institutions” including four preplanned questions/discussion areas. The first question: “*How does your identity/positionality influence your involvement in research/knowledge creation based on Indigenous knowledge?”*, with following discussion areas focusing on: *“What has been your experience working within a colonial institution considering...*: ‘*interactions with the institution’, ‘interactions with colleagues’* and ‘*interactions with journal peer-review systems?’”*.

The topic of “Identity and Colonial Institutions” was explored through a series of successive talking circles, each audio-recorded, and averaging 55 minutes duration. Participant groups each began at a different whiteboard station. At each station, a set of questions related to the rigorous use of Indigenous knowledge systems and methodologies within Indigenous research was posted. Groups discussed the topic and recorded their ideas on the whiteboard using notation, flowcharts, and imagery. Once a group had completed its discussion at a given station, it rotated to a new whiteboard featuring a different set of questions. Before beginning the next discussion, the group first reviewed the notes left by the previous group, allowing them to build upon earlier ideas. This process continued until all three groups had visited and contributed to each of the thematic whiteboards. Photographs of the evolving whiteboard notes were taken after each group discussion, creating a visual record of the layered thinking and providing a data source for later reflection and analysis.

After the rotation was complete, the first group returned to its original whiteboard to synthesize key ideas across all contributions. This brief synthesis circle, also audio-recorded (5 minutes in duration), identified prominent themes and insights from the cumulative notes. To conclude the activity, a final large group talking circle (31 minutes in duration) brought all participants together for collective reflection. The first part of this concluding discussion (approximately 9 minutes) focused specifically on *Identity and Colonial Institutions in Indigenous Science*.

### Data analysis

Audio-recorded talking circles were transcribed verbatim and two members of the team (ally graduate student and First Nations faculty researcher) independently undertook inductive thematic analysis as outlined by Braun and Clarke [27]. The whiteboard discussion photos were used to strengthen interpretation [28]. A visual representation of identified themes was developed and shared with participants to facilitate team member feedback and increase accessibility of results [29,30].

### Recommendation development

Two Métis faculty researchers (co-first authors) reviewed the themes alongside the transcripts and developed recommendations for supporting Indigenous science in Western, colonial-grounded academic institutions. The recommendations developed are inherently reflective of their experiences, identities, and cultural backgrounds. These recommendations were then shared with and thoughtfully reviewed by participants and updated to reflect feedback, perspectives, and experiences of participants.

## Results

**Fig 1:**
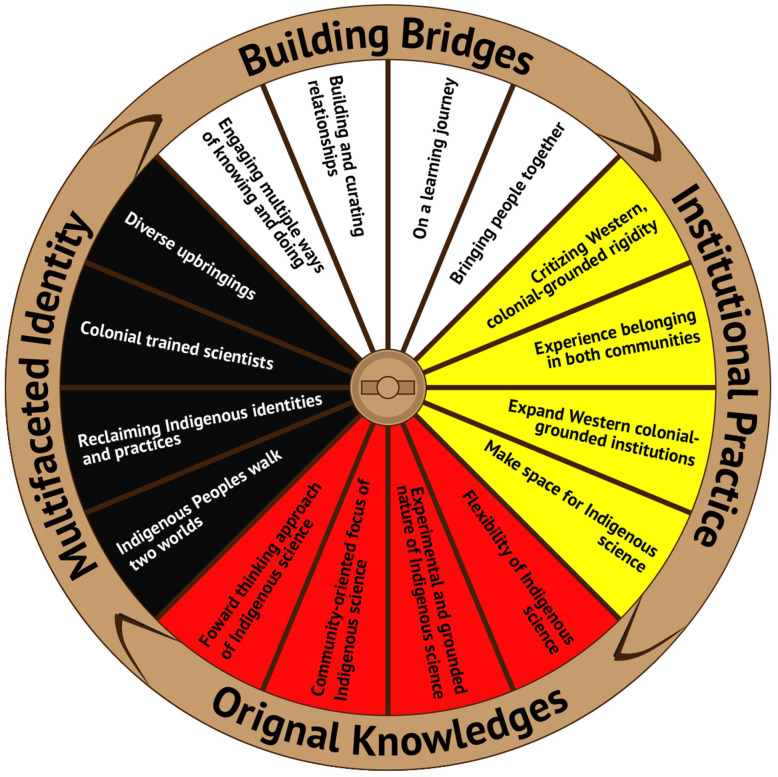
Themes generated from “Identity and Colonial Institutions” discussions among Elder/knowledge helper/knowledge keepers, community member, Indigenous researchers, trainees, and allies in January 2019: 1) Building Bridges; 2) Institutional Practice; 3) Original Knowledges; and 4) Multifaceted Identity.

Four themes were identified on the topic of “Identity and Colonial Institutions”, as outlined in Fig 1. The *Building Bridges* theme centred on topics of: i) engaging multiple ways of knowing and doing; ii) building and curating relationships; iii) on a learning journey; and iv) bringing people together. Conversations around *Institutional Practice* brought forth ideas of: i) criticizing Western, colonial-grounded rigidity; ii) the need for Indigenous Peoples to experience belonging within both Western, colonial-grounded institutions and their respective Indigenous communities; iii) discussing opportunities to expand and enhance Western, colonial-grounded institutions through engagement of Indigenous science; and iv) recognizing the need to make space Indigenous science in Western, colonial-grounded institutions. The theme *Original Knowledges* reflects discussions on recognizing the: i) flexibility of Indigenous science; ii) experimental and grounded nature of Indigenous science; iii) community-oriented focus of Indigenous science; and iv) forward-thinking approach of Indigenous science. Discussions around identity for Indigenous Peoples in Western, colonial-grounded institutions focused on *Multifaceted Identity*, recognizing: i) Indigenous Peoples walk two worlds; ii) the importance of reclaiming Indigenous identities and practices for Indigenous Peoples in Western, colonial-grounded institutions; iii) Indigenous Peoples’ experiences as colonial trained scientists in colonial institutions; and iv) the diverse upbringings within and across Indigenous Peoples.

As described on Table 1, recommendations for academic institutions, funding bodies, peer-reviewed journals, and all academic institutions, organizations, and entities to support Indigenous science were developed around four central actions: 1) Embedding respectful and authentic support throughout Western, colonial-grounded institutions; 2) Acceptance, endorsement, incorporation, and education among the broader research community; 3) Prioritizing and valuing Indigenous knowledges, processes, and contributions; and 4) Privileging multiple worldviews. Enacting these recommendations to authentically support Indigenous knowledges and Indigenous science in Western, colonial-grounded academic institutions will require a fundamental shift to the way knowledge is conceptualized, valued, and institutionalized within Western academic systems.

**Table 1:** Actions and recommendations to support flourishing of indigenous science in Western, colonial-grounded institutions.

Action	Recommendations	In Practice
1. Embedding respectful and authentic support throughout Western, colonial-grounded institutions	1.1. Genuine research program funding applications and budgets	Grant applications and long-term research plans (i.e., 5-year grant application) need to allow for or consider unstructured or less clearly articulated plans for later years, to honour the importance of community voice and needs, and the partnership journey rather than a planned output. Research structures (i.e., grant funding within budget categories) need to be flexible and accommodate this partnership journey, recognizing research plans may change after being initiated.
	1.2. Equitable support and respect for Indigenous community-led research	Required demands for holding and administering grants without support and financial in-kind to undertake these requirements leaves Indigenous communities with undue burden in undertaking Indigenous community-led research. This creates inequality compared to research through Western, colonial-grounded institutions. Indigenous communities need access administrative funding attached to tri-council grants either directly, or through hybrid models such as through the NEIHRs to reduce this inequitable barrier.
	1.3. Honouring the importance of relationships and partnerships	Recognition of time required to build relationships and engage in respectful Indigenous research processes is required. Harshly specific and rigid timelines for funding applications, graduate degrees, new investigator definitions, and tenure processes, and recognition/expectation of research “outputs” are not conducive to the inherent relationality of Indigenous research. Longer supports and extended standard timelines may be needed.
	1.4. Supporting Indigenous talent and community-led initiatives	Stable support, expectations, and criteria for researchers, grant programs, and trainee funding are required. Changing eligibility, requirements, deadlines, opportunities, expectations, or funding levels after initiatives have started (i.e., grant applications, research programs, community partnerships, etc.) can cause undue stress and challenges for Indigenous communities. When changes are absolutely essential, ample notice and joint conversation to prepare and plan for changes will enhance Indigenous communities’ opportunities for successful continuation and participation under changed circumstances.
	1.5. Eliminating administrative burdens for community member participation in research and funding processes	Engaging community members as researchers within funding applications currently requires community members to complete colonial processes to be recognized as a part of the research team, creating barriers to full partnership and recognition of community members. Removing these account and form creation requirements for community partners will better support community member participation in applications.
2. Acceptance, endorsement, incorporation and education among the broader research community	2.1. Establishing, incorporating, and valuing spaces where Indigenous Peoples “see” themselves	Support of Indigenous talent and communities’ participation in research requires “seeing” themselves within the institution. Universities and funding agencies need to be spaces where Indigenous Peoples feel welcome, are accepted and treated with respect, and where Indigenous presence and processes are infused throughout all spaces (i.e., the physical, conversational, and electronic/online spaces).
	2.2. Creating opportunities for developing authentic relationships and partnerships	Supporting all researchers, regardless of their history of engagement with Indigenous communities, to build relationships with Indigenous communities will facilitate authentic relationship-building and eventual research partnerships across varied disciplines. Broad support and engagement of Indigenous research throughout all aspects of institutions and research will facilitate enhanced recognition, value, and support of Indigenous ways of knowing, being, and doing.
	2.3. Acceptance, incorporation, and appropriate ongoing, lifelong training for reviewers, researchers, trainees, and other research personnel	Acceptance, incorporation, and appropriate ongoing, lifelong training is needed to support reviewers (i.e., grant, manuscript, abstract), researchers, trainees, and other research personnel to have the requisite knowledge and experience to conduct, evaluate, and review Indigenous research (i.e., research methods courses, research workshops, and/or training modules). Knowledge of Indigenous science among all researchers will improve all research, Indigenous and non-Indigenous alike.
	2.4. Acceptance, incorporation, and recognition of community-specific education, teachings, learnings, and training	“Training” in an Indigenous context (see 2.3) includes a broader range of activities than typically recognized in Western, colonial-grounded institutions, including sitting with Elders^†^, storytelling, conversations, visiting, ceremony (where and when appropriate), and land-based activities. Training is specific to individual Indigenous communities and cannot be applied in broader “pan Indigenous” approaches. While some general training can be provided by national initiatives, specific training and learning from the community is necessary before undertaking research with Indigenous communities.
3. Prioritizing and valuing Indigenous knowledges, processes, and contributions	3.1. Recognizing and prioritizing Indigenous Elders^†^ as experts of Indigenous knowledges and ways of knowing, being, and doing.	Prioritizing and valuing traditional education and training to achieve roles of Elders^†^ as higher than what university-trained scholars (i.e., PhD or researcher) hold. Current power imbalances must be disrupted to eliminate the hierarchy of Western, colonial-grounded research.
	3.2. Inclusion and prioritization of Indigenous experts in research review processes	Respectful and appropriate inclusion of Indigenous experts (see 2.1) as part of research review processes (i.e., grants, manuscripts, articles), requires consideration of competing obligations, responsibilities, and roles Indigenous Peoples hold, particularly to home communities. Equitable compensation needs to be incorporated, while supporting Indigenous experts to maintain traditional values, principles, and processes without compromise within the Western, colonial-grounded institution and its colonial processes.
	3.3. Respectful and authentic relational research partnerships	Respectful and authentic relational research partnerships require timelines and expectations prioritizing Indigenous community members’ multiple roles and responsibilities. Support, reviews and input requests on colonial processes (i.e., grant applications, ethics applications) need to provide sufficient time for the community to complete tasks without undue burden.
	3.4. Prioritizing Indigenous researchers, experts, and trainees in research processes	Indigenous researchers, experts, and trainees hold knowledge and experience from both Indigenous and Western education systems. This additional experience held exclusively by Indigenous researchers, experts, and trainees should be valued and uplifted in research processes. Indigenous researchers should be included as authentic members of research teams, and Indigenous trainees prioritized for Indigenous science research.
	3.5. Authentic relationships, projects, and partnerships	Significant and unhurried consultations, discussions, and conversations with Indigenous communities and members are needed before decisions and directions are determined.
	3.6. Incorporating and valuing Indigenous research as a journey of partnership	Indigenous research is mutual partnership journey. Research plans and measures of “success” need to incorporate this research approach. Recognition of outputs that matter to Indigenous communities (i.e., relationship building activities, knowledge sharing activities, community events) as equivalent to Western metrics (i.e., publications, grants) will support Indigenous talent and research.
4. Privileging multiple worldviews	4.1. Endorsement and incorporation of Indigenous ownership of knowledges	Endorsement and incorporation of appropriate approaches, protocols, practices, and evaluation metrics and methods requires processes built to honour Indigenous communities as genuine “gatekeepers” of Indigenous knowledges and science. Indigenous Nations, communities, and Peoples allow researchers to be part of community research and knowledge, not researchers allowing or enticing communities to be part of their research program.
	4.2. Breaking down the “hierarchy” in determining research agendas	Community needs and voices are prioritized as driving forces in research. Communities have self-determination to decide, without undue influence, participation in mutual research partnerships with researchers/institutions, prioritizing impacts and value for their community. It is inappropriate for funders, institutions, and researchers to hold all the power, particularly when funding has already been obtained and can be perceived as coercive to lure Indigenous communities into pre-planned research initiatives.
	4.3. Recognizing, respecting, and incorporating distinctiveness of Indigenous communities	Inclusion, implementation, and appropriateness of spirituality and ceremony within Indigenous research processes will be specific to unique projects, participants, communities, and relationships. Undertaking and reporting research with Indigenous communities needs to engage distinctions-based approaches, to recognize and reflect these diversities.
	4.4. Research processes, structures, and supports need to consider the unique situations and needs of the community	Research processes, structures, and supports (i.e., ethics approvals, research protocols, community research agreements, etc.) need to be flexible to implementation and actualization of diverse circumstances.

† Elder and Knowledge Keeper are current academic terms describing Indigenous individuals who hold, practice, transmit, and support Indigenous knowledges, teachings, cultural practices, and respected positions reflective of their knowledge and expertise within their specific Indigenous community. Within and across Indigenous communities, varied terms are preferred, such as Old Ones, Holy Ones, or knowledge holders. Terms used should reflect specific community practices, cultures, and self-identification.

***Action 1. Embedding respectful and authentic support throughout Western, colonial-grounded institutions*** requires a fundamental shift from conventional academic approaches. Engaging in and supporting Indigenous science means valuing and recognizing Indigenous knowledge systems as equal to those rooted in Western colonial traditions. Authentic support involves a rethinking of how research is defined, practiced, and evaluated within institutional structures that have historically marginalized non-Western epistemologies. Supporting Indigenous research and talent requires processes, timelines, and structures that take into consideration Indigenous ways of knowing, being, and doing, guided by five recommendations outlined in Table 1.

Grant funding for Indigenous science needs to honour the importance of community voice and allow for community diversity and community-specific needs (recommendation 1.1). Planned research activities over longer term grants (i.e., 5-years) may need flexibility to adapt to and accommodate changing community needs, priorities, and resources beyond the initial years of a project. Similarly, subsequent studies planned within grant application need flexibility to adapt to initial study findings and changing community needs. Research structures, such as grant funding within specified categories, also need flexibility to accommodate the partnership journey. For example, community staffing changes such as community nurses no longer being available to help with data collection, may necessitate funding shifted across budget categories to hiring a community member or nursing student to continue data collection.

Tri-council research funding provides Western, colonial-grounded institutions with research overhead funding to support grant and research administration. However, this administrative support is not always extended to Indigenous communities or organizations holding research funds, potentially placing undue burden on Indigenous community-led research. Equitable support and respect for Indigenous community-led research (recommendation 1.2) requires equitable administrative funding, or access to supports funded by such administrative funding to accompany research grants held by Indigenous communities or organizations. One potential solution incorporates hybrid models, such as the Network Environments for Indigenous Health Research (NEIHR), providing institutional support for Indigenous community-directed and held funding [31].

Honouring the importance of relationships and partnerships (recommendation 1.3) requires recognizing time investments and commitments needed to build and maintain respectful partnerships with Indigenous communities. Graduate degree requirements, new investigator definitions, and tenure, promotion, and merit processes do not always support authentic relationships with Indigenous communities. For example, graduate students’ graduate degree time and funding limitations do not consider the considerable time students spend building partnerships with Indigenous communities before research initiation. Such extra time commitments may extend beyond funding eligibility and require additional tuition payments.

Faculty working in areas of Indigenous science also face inequitable burdens that contradict the inherent relationality of Indigenous science. Pressure to produce research “outputs” focused on Western metrics (i.e., publications, grants, citations) often conflicts time-intensive, relationship-based approaches required for meaningful engagement with Indigenous communities. Similarly, faculty working in areas of Indigenous science face inequitable evaluation of their work for promotion through academic ranks, merit/salary increase evaluations, and consideration for advancement to administrative positions. This misalignment creates tensions between academic expectations and the ethical responsibilities of conducting Indigenous-led research.

Harshly specific, often tight, and rigid timelines required for research funding applications, create similar undue burdens on Indigenous communities, and disrespectfully pressures Indigenous communities to support funding applications under tight timelines. Consistent and predictable funding provides opportunities for research partnerships to flourish and to develop funding applications when appropriate for Indigenous communities and research partnerships. Similarly, stable support, expectations, and criteria for researchers, grant programs, and trainee funding are required (recommendation 1.4). Changes to eligibility requirements, deadlines, funding opportunities and expectations, or levels of funding after funding calls have been advertised, and particularly after planning grants for larger initiatives have been awarded, create undue stress and challenges for both Indigenous communities and researchers working in areas of Indigenous science. When changes are essential, ample notice and joint conversations with Indigenous communities and researchers in areas of Indigenous science are needed to enable planning and adjustment to changes.

The inclusion of community members, leaders, and experts as research team members for funding applications and publications often requires administrative hurdles to fit colonial processes. Eliminating administrative burdens for community member participation in research and funding processes is needed to respectfully and fully engage community members in Western, colonial-grounded research incorporating Indigenous science (recommendation 1.5).

***Action 2. Acceptance, endorsement, incorporation, and education among the broader research community*** are essential to cultivating a research environment inclusive of Indigenous science. This entails actively learning about and integrating Indigenous perspectives and committing to ongoing education across disciplines. Respectful engagement demands not only inclusion but also transformation—where Indigenous knowledges are not simply added to existing frameworks but contribute to reshaping them. Full support and engagement of Indigenous research requires critical change beyond current Indigenous talent and research partnerships, including change to the broader research community, supported by four recommendations.

Including Indigenous science and talent within Western, colonial-grounded institutions begins with creating welcoming spaces where Indigenous talent feels included and “sees” themselves. Western, colonial-grounded institutions (including universities, funding agencies, and publishing bodies) need to be spaces, both physical and online, where Indigenous Peoples feel welcome and respected (recommendation 2.1). The presence of Indigenous Peoples and processes infused throughout all spaces is needed to fully welcome and incorporate Indigenous science and talent within Western, colonial-grounded institutions. Transforming Western, colonial-grounded institutions to include Indigenous science should also support development of authentic relationships and partnerships (recommendation 2.2). By creating and supporting opportunities to build relationships with Indigenous communities among researchers, regardless of experience engaging with Indigenous communities, authentic relationship-building and partnerships across varied disciplines can be facilitated. Supporting partnership development among researchers without experience partnering with Indigenous communities, particularly among disciplines where Indigenous science engagement is limited, will provide opportunities to share knowledge systems, enhance recognition and valuing of Indigenous science, and expand engagement of Indigenous science.

Acceptance, incorporation, and appropriate ongoing, lifelong training and education beyond individuals engaged in Indigenous science are needed to fully support Indigenous science in Western, colonial-grounded institutions. Knowledge of Indigenous science is needed among all researchers and individuals engaged in grant, manuscript, and academic review processes (recommendation 2.3). A basic understanding of Indigenous science will enable reviewers to identify when Indigenous science and Indigenous community partnership should be considered, such as projects where Indigenous Peoples are overrepresented. Further, Indigenous science is valuable and applicable across all disciplines. Recognizing this value supports innovation and advancement across all fields.

For individuals engaged in Indigenous science, community-specific training is required (recommendation 2.4). Understanding community-specific protocols and needs is essential to authentically engage research with Indigenous Peoples. Recognition of Indigenous and community-grounded training methods among the larger Western, colonial-grounded institutions is needed to support community-specific training requirements.

***Action 3. Prioritizing and valuing Indigenous knowledges, processes, and contributions*** is paramount. Indigenous science must not be viewed as central and essential to knowledge production. This prioritization requires systemic changes in how research agendas are set, contributions are assessed, and Indigenous voices are positioned within scholarly discourse. Indigenous learning and education processes have existed longer than Western colonial-grounded institutional systems. Privileging and prioritizing traditional knowledge systems and processes is supported by six recommendations.

Prioritizing and valuing Indigenous knowledges, processes, and contributions as equal to Western knowledge systems requires prioritizing and valuing of experts from both systems (recommendation 3.1). Within Indigenous knowledge systems, experts are individuals who are given titles such as Elder, bestowed by their community. Employing and engaging these experts at Western, colonial-grounded institutions is needed. Further inclusion of Indigenous knowledge system experts in Western processes that engage Indigenous science is also needed (i.e., grant, manuscript, case file reviews; recommendation 3.2). Recognizing the inherent responsibilities and commitments Indigenous Elders and community leaders have to their communities, including traditional values, principles, and processes requires consideration of time commitments (recommendation 3.3). Sufficient time for requests of support, reviews, or input requires consideration of community responsibilities held by community leaders and experts. Subsequently, equitable compensation and considerations of time requests from Elders are also required. Valuing Indigenous Peoples’ experience, knowledge, and expertise in Indigenous science requires recognizing additional experience, knowledge, and expertise of Indigenous Peoples in Western, colonial-grounded institutions, including Indigenous researchers, trainees, and staff (recommendation 3.4). Valuing this experience, knowledge, and expertise includes prioritizing Indigenous researchers, trainees, and staff for activities and initiatives engaging Indigenous science within Western, colonial-grounded institutions.

Transforming Western, colonial-grounded institutions to include Indigenous science requires authentic relationships, partnerships, and projects that respect the diverse demands, needs, and commitments of Indigenous communities and Peoples (recommendation 3.5). Shared decision-making authority and leadership in projects incorporating Indigenous science requires respectful and unhurried discussions, conversations, and consultations with Indigenous communities and experts before decisions and directions are determined. Unhurried relationships provide Indigenous communities and partners sufficient time to consider potential projects, grant applications, publications, conference presentations, and other research activities and outputs. Engaging Indigenous science in Western, colonial-grounded institutions requires incorporating Indigenous processes and values. Authentic relationships reflecting Indigenous values bring together Indigenous science and Western, colonial-grounded institutions in collaborative journeys. These partnerships emphasize ongoing, long-term commitments to working together as equals for mutual change and shared success for Indigenous communities and Peoples, as well as Western, colonial-grounded institutions (recommendation 3.6). Valuing this partnership journey requires privileging outputs and measures of success important to partnering Indigenous communities, such as valuing community knowledge sharing events as equal to Western publications and conference presentations, attaining community approval of a project equivalent to a grant application, or Elder’s acceptance of tobacco representing high impact factor publications.

***Action 4. Privileging multiple worldviews*** means recognizing the legitimacy of diverse ways of knowing, being, and doing—beyond Western epistemologies. Respectful and meaningful engagement with Indigenous science demands that multiple knowledge systems be regarded as equal in value and significance. Supporting Indigenous science thus involves creating space for epistemic pluralism within research institutions and practices. Distinct Indigenous Nations and communities are varied and vast, each with their own traditions, culture, knowledges, and ways of knowing, being and doing. The uniqueness of each community and its people underscores the range of worldviews across different research projects. Mutual respect and privileging of Indigenous communities’ ways of knowing, being, and doing requires a shared research journey partnering with Indigenous communities as equals, rather than “petri dishes” to be studied, supported by four recommendations.

In contrast to Western academic ways of approaching intellectual property, copyright, and publication, Indigenous science requires endorsement and incorporating Indigenous ownership of their knowledges (recommendation 4.1). While numbers, types, and equivalencies of publications for academics engaged in Indigenous science may be impacted, recognition of Indigenous ownership is paramount. Importantly, recognizing Indigenous ownership requires approaches, endorsement, protocols, practices, and evaluation metrics and methods built around Indigenous communities and Peoples as the gatekeepers of Indigenous science. Indigenous communities invite academics to incorporate or explore their traditional knowledges, practices, and cultures within research contexts. Academics must not push Indigenous communities to be part of planned (sometimes already funded) research programs. Alongside Indigenous ownership and decision-making authority, community self-determination, decision, and direction must be incorporated (recommendation 4.2). Indigenous communities determine their participation in research and direct decision-making for research projects and funding. When researchers already hold grant funding and recruit communities to participate in researcher-driven projects, communities may feel coerced into participating. Using funding or other enticements to lure Indigenous communities to partner on research projects undermines the principles of Indigenous autonomy, ownership of their knowledges, and self-determination.

Research engaging Indigenous science needs to be flexible enough to meet diverse community structures and appropriate cultural protocols of the specific community (recommendation 4.3). Participants are also at different places in spiritual and cultural journeys, potentially necessitating multiple cultural protocols within a single project. While research processes, ethics protocols, and community approval processes and research agreements may be required for projects incorporating Indigenous science, specific requirements need to be flexible to diverse partnering communities (recommendation 4.4). Governing structures vary across Indigenous communities, requiring varying approval and partnership processes. Recognizing distinctiveness of Indigenous communities also requires distinctions-based research, where results are reported by Indigenous identity, Nation, or community, to reflect this diversity. The spectrum of Indigenous science also includes research where Indigenous Peoples are overrepresented (i.e., land-based educators, traditional games facilitators, educators in schools incorporating cultural programs). These varied community structures will require community partnership/approval, and ethics approval; however, specific process and subsequent institutional requirements need to be flexible depending on the specific community.

## Discussion

This study explored fundamental actions needed to support flourishing of Indigenous science within Western, colonial-grounded institutions. Four themes identified from talking circles informed development of four major actions for Western, colonial-grounded institutions to support Indigenous science: *Respectful and Authentic Support*; A*cceptance, Endorsement, Incorporation and Education Within the Broader Research Community*; *Prioritizing and Valuing Indigenous Knowledges, Processes, and Contributions*; and *Privileging of Multiple Worldviews*. Our study’s primary contribution to the literature articulates concrete actions and recommendations to support the flourishing of Indigenous science within Western, colonial-grounded institutions, advancing efforts toward decolonial Indigenization [24]. These recommendations apply across disciplines, with potential to strengthen diverse disciplines from geography to natural and social sciences, engineering, medicine, education, and technology studies. The historical systemic exclusion of Indigenous science within research institutions is grounded in “racist and colonial hegemony of Western science” (p.88) [1]. Incorporating Indigenous science into research institutions strengthens all areas of understanding, advancing knowledge. Improved learning outcomes identified when Ethno science is incorporated into educational contexts further supports this strengthening of understanding and advancing knowledge by supporting flourishing of Indigenous science within Western, colonial institutions [3,32,33]. These recommendations are grounded in the context of Indigenous science in what is now known as Canada, and strongly influenced by policies such as the TCPS 2 Chapter 9 research guidelines [34]. In advancing knowledge, toppling the hegemony of modern colonial science globally provides opportunities to incorporate multiple, distinct knowledge systems and forms of science. As such, some form of these recommendations may be applicable outside of Canada, and may be relevant to support the inclusion of other forms of science outside of modern colonial and Indigenous sciences.

This work, and experiences of Indigenous and ally participants, builds on key concepts and approaches for engaging Indigenous science within Western, colonial-grounded institutions previously identified in the literature. *Building Bridges* between Indigenous science and Western, colonial-grounded knowledge and knowledge systems, and changing *Institutional Practices* to endorse, prioritize, and privilege *Original Knowledges* and *Multifaceted Identity* requires collaboration among Indigenous Peoples, communities, Nations, and science with Western, colonial-grounded institutions, non-Indigenous people, and federal, provincial, and local governments. Imperial underpinnings of research as understood from a Western, colonial perspective discredit traditional Indigenous science through ‘regimes of truth’, and concepts of ‘discovery’ and ‘claiming’ [35]. Our recommendations to recentre Indigenous science as legitimate, sovereign, and relational systems of inquiry alongside Western, colonial research are consistent with foundational calls for decolonizing research by Linda Tuhiwai Smith [35]. Such efforts align with Nehiyaw ethicist and researcher, Willie Ermine’s concept of “Ethical Space”, envisioning dialogue between distinct worldviews and creating space for mutual respect and shared learning [36]. A genuine partnership between these epistemologies requires working within “Ethical Space” to achieve a cooperative spirit and create new currents of thought, expanding knowledge, research, and innovation [36].

This vision also resonates with Hodinöhsö:ni’ Two Row Wampum-Covenant Chain, and associated research partnership principles developed by Six Nations of the Grand River knowledge keeper Rick Hill and settler scholar Daniel Coleman. Their principles emphasize the tradition of “place-conscious ceremony” to honour the sacred space between entities [37]. Our recommendations also build on research principles derived from Two Row Wampum Belt, including “Relationships are dialogical” in valuing differences of both Indigenous science and Western, colonial-grounded knowledge and knowledge processes, and “Equity within distinctiveness” which calls for relationships that recalibrate authority and leadership recognizing individual differences in experiences, histories, and grounding [37]. Further, “Sharing knowledge, not owning it” directly challenges Western ownership claims over Indigenous science. Our recommendations also reflect “Internal pluralism and diversity” resisting assumptions of homogeneity within both Indigenous and Western, colonial-grounded knowledge and knowledge systems [37]. Bringing together Indigenous science with Western, colonial-grounded knowledge and knowledge processes provides opportunities for “Etuaptmumk (Two-Eyed Seeing)” [38]. The concept of “Etuaptmumk”, described by Mi’kmaq Elder Albert Marshall offers a framework drawing on strengths of both systems to benefit all [38]. Our recommendations aim to contribute to this evolving partnership and cooperative spirit aligned with respective Indigenous Nations’ research frameworks.

Relationship-building is a recurring theme among recommendations to support Indigenous science in Western, colonial-grounded institutions [39], and a CRCC key priority [40]. However, our recommendations extend further, addressing power imbalances structuring these relationships as recommended by Gilbert et al. [41]. For example, recognizing Indigenous ownership of knowledges (Recommendation 1.1) and dismantling hierarchical control over research agendas (Recommendation 1.2) require relationships and fundamental changes to how research is conducted and governed, placing authority with Indigenous communities, rather than the academy. Such shifts align with recommendations for Indigenous self-determination in research priorities as outlined by NSERC [42] and the CRCC [43]. Recommendation 4.2 reflects this emphasis on Indigenous self-determination in research. Our relationship recommendations also highlight respectful and authentic relational research partnerships (Recommendation 3.3) and authentic relationships, projects, and partnerships (Recommendation 3.5). Authentic and respectful relationships require flexible timelines and processes, adjusting grant application cycles and research workflows to accommodate long-term Indigenous community and Western, colonial-grounded institution engagement (Recommendation 3.6), consistent with “Etuaptmumk” [38].

A central NSERC strategy is equitable funding and training access for Indigenous students and researchers [42]. Our recommendations move beyond equitable access to emphasize prioritization of Indigenous researchers, experts, and trainees in project leadership (Recommendation 3.4). Although prioritizing Indigenous talent has not been specifically endorsed by the CRCC, their strategic goals include increasing Indigenous representation within federal granting agencies and integrating Indigenous perspectives into high-level decision-making [43].

Our call for equitable support and respect for community-led research (Recommendation 1.2) is grounded in evidence of systemic barriers to Indigenous self-determination in research, including rigid funding eligibility criteria and institutional structures that marginalize Indigenous knowledges [31]. Hybrid models such as those supported by the NEIHRs could support our Recommendation 1.5, eliminating administrative burdens and increasing accessibility for community participation [31].

The CRCC identified revising eligibility requirements as important commitments to ensure equitable access to research funding and infrastructure support for Indigenous organizations and create effective tools and resources to build and strengthen understanding and user-friendliness of granting agency programs [43]. Our recommendations for equitable support and respect for Indigenous community-led research (recommendation 1.2), and genuine research program funding applications and budgets (recommendation 1.1) similarly aim to ensure equitable research funding and infrastructure access, and strengthen user-friendliness of granting agencies including application processes [43]. Importantly, our recommendations provide more specific direction, potentially moving beyond CRCC objectives [43]. Equitable access to research funding and infrastructure support needs to include administrative funding and supports (recommendation 1.2), and grant timelines that enable *equitable* research activities (recommendation 1.1), such as longer or more flexible timelines and budgets [43]. Further, consistent with our recommendation 1.5, user-friendliness of granting agency programs may not be sufficient to accomplish eliminating administrative burdens for community member research and funding processes participation [43].

Australian recommendations to support Indigenous graduate students highlight training and education for all academic staff, similar to our recommendation 2.3, and inclusion of Indigenous experts in academic processes for graduate students, in line with our recommendation 3.1 [44]. Supporting Indigenous talent (recommendation 1.4) is improving in Canada, such as targeted trainee funding from the Heart and Stroke Foundation of Canada [45], or expanded funding or application quotas for Indigenous trainees with SSHRC, CIHR, and NSERC [46,47]. Australian recommendations reach further, suggesting establishment of an database of Indigenous academics who can supervise Indigenous students [44]. Establishment of such databases may help identify potential Indigenous supervisors; however, limiting potential supervisors to Indigenous academics creates gaps for institutions and disciplines with few Indigenous academics, highlighting important Indigenous faculty growth needs. Easy, online identification of Indigenous academics may help facilitate identifying potential supervisors for Indigenous graduate students.

Accountability in respecting Indigenous ethics and protocols, and identifying benefits of research projects for Indigenous communities are also strategies identified by NSERC [42]. The CONSIDER statement for reporting Indigenous health research also identifies needs for Indigenous community benefits, particularly engaging capacity building [48]. Our recommendation 4.4 similarly considers unique community needs and situations and builds on community-specific process recommendations. However, greater change is needed, highlighting Indigenous science in research as a journey of partnership (recommendation 3.6), reflecting long-term commitments to research partnerships, and relationships built on mutual respect, shared benefits, and joint leadership with shared decision making. A review by Alcock et al. found Memoranda of Understanding (MOUs) for research ethics within Indigenous science are generally not shared publicly and may not be commonplace, highlighting a need for greater awareness of such tools, and incorporation of MOUs as a possible approach to Indigenous science [49]. More recently, Memoranda of Relational Understanding (MORU) are being engaged recognizing relationality as fundamental to relationships with Indigenous communities. Adams and Faulkhead [50] note “there is not and cannot be a step-by-step guidebook to community partnerships” (p.1016), highlighting the need for flexibility in academic protocols and processes (recommendation 4.3). Since each community partnership looks, functions, and is experienced differently, processes around partnerships need to be flexible to meet community-specific needs and capacities. Previous recommendations identify necessity in being cognizant of distinctive differences between Indigenous Nations and communities [41]. Beyond awareness of distinctiveness, individuality and distinctiveness of Indigenous communities, cultures, and knowledge systems requires recognizing, respecting, and incorporating distinctiveness of Indigenous communities in academic processes (recommendation 4.3).

Consideration of unique situations and needs of community partners are required throughout research processes, structures, and supports provided and implemented (recommendation 4.4). Further, we recommend acceptance, incorporation, and recognition of community-specific education, teachings, learnings, and training (recommendation 2.4) where individuals gain and share knowledge and experience about the history, protocols, culture, and ways of engaging in respectful relationships specific to the partnering community. Together, these recommendations represent an important aspect of Indigenous science, meeting communities where they are at.

Recommendations for community engagement in Indigenous health research highlight determining needs and roles for the community in research [39]. Rigidity in research processes, such as institutional ethics applications and grant application requirements create barriers for communities who are structured differently, or who have limited or diverse experiences engaging in research. Institutional training and requirements for research protocols and ethics also need to consider distinctions-based approaches. Many ethics boards follow The First Nations Principles of OCAP^®^ as a checklist or guideline for research ethics with Indigenous communities [51]. These principles, however, are specific to First Nations. Métis [52] and Inuit [53] perspectives on research ethics and data sovereignty, Data Governance Principles, or community-specific protocols need to be recognized and appropriately. Beyond institutional processes, distinctions-based Indigenous community research and reporting is needed, recognizing and reflecting the diversity across and within Indigenous Nations, communities, and Peoples [54].

A disconnect between Western, colonial-grounded institutional processes and Indigenous methods, particularly for tenure evaluation was recently reported, advocating for changes to evaluation metrics in academia [55]. This call for evaluation metric changes has similarly gained traction in Western, colonial-grounded institutional practices through the San Francisco Declaration on Research Assessment (DORA) [56]. Our recommendation 1.3, including revised timelines and “outputs” considers multiple research metric methods and impacts on policy and practice [56], but extends beyond DORA to consider real life impacts and benefits for partnering Indigenous communities and adjusts expectations around metrics and publications to reflect time commitments required for Indigenous science.

A key aspect of our recommendations considers Western, colonial-grounded institutions in general, where Indigenous science is not engaged. Specifically, recommendation 2.3 calls for acceptance, incorporation, and appropriate ongoing, lifelong training for reviewers, researchers, trainees, and other research personnel for all individuals involved in activities where Indigenous science could arise. This includes grant and manuscript reviews, teaching evaluations, research methods courses, etc. outside of Indigenous science, to enable requisite knowledge and ongoing lifelong training for all faculty to know if Indigenous science will, should, or may be appropriately implemented across all academic contexts. Further, recommendation 2.2 calls for acceptance, incorporation, education, and training for Western, colonial-grounded institutions in general to provide opportunities to expand understandings of Indigenous science and build relationships among individuals and disciplines where Indigenous science has traditionally not been engaged. A key mechanism among the CRCC is offering funding opportunities to support relationship building with Indigenous Peoples [43]. Gaining preparation and learning about Indigenous Peoples and Indigenous research ethics is an introductory stage, providing acceptance, incorporation, and appropriate ongoing, lifelong training for all academics and trainees [39].

Incorporation of Indigenous science into Western, colonial-grounded institutions is consistent with previous recommendations to support Indigenous Peoples’ education [12,23,57,58]. We recommend establishing, incorporating, and valuing safe spaces for Indigenous Peoples, and the ability for Indigenous Peoples to “see” themselves, their cultures, and histories across all aspects of Western, colonial-grounded institutions including content and courses, processes and systems, supports, and environments, including physical, experienced, and perceived spaces.

New recommendations from this study focus on stable and predictable funding opportunities (recommendation 1.4), which supports relationship building and self-determination in developing funding applications and designing research projects. Additionally, flexible research project timelines and flexibility for spending grant budgets across grant application categories are needed to support and adapt to community needs and research findings (recommendation 1.1).

A clear strength of these recommendations is their collaborative and evidence-based development from talking circles among a diverse group of Indigenous Elders/knowledge helper/knowledge keepers, community members, researchers, and trainees, as well as ally researchers and trainees. Diverse participant experiences from multiple geographies, institutions, funding bodies, career stages, and disciplines, with diverse Indigenous Nations, communities, governing bodies, and organizations provides a strong foundation for development of these recommendations. Incorporation of Indigenous science within Western, colonial-grounded institutions continues to evolve, and experiences among Indigenous communities and Peoples in research may differ. Additional actions and future recommendations to support Indigenous science in Western, colonial-grounded institutions may shift and change over time. Further, a lack of Inuit participation in this project highlights a gap in knowledge. Wholistically moving these actions forward will require input of Inuit experiences and recommendations, with regular reflection and revision of future steps and recommendations. Future projects to further this work should specifically engage Inuit perspectives to wholistically develop future recommendations.

## Conclusions

Calls for authentic and respectful incorporation of Indigenous science into Western, colonial-grounded institutions continue, recognizing the benefits of bringing together diverse worldviews. Incorporating Indigenous science into Western, colonial-grounded institutions requires transformation to Western, colonial-grounded institutions’ view and valuing of Indigenous knowledges, values, and ways of knowing, being, and doing.

Actions are needed to ensure *respectful and authentic support*, *prioritizing and valuing Indigenous knowledges, processes, and contributions*, *respectfully and authentically incorporating acceptance, integration, and education within the broader research community*, and *privileging of multiple worldviews*. These efforts are essential for meaningful and respectful incorporation of Indigenous science into Western, colonial-grounded institutions. Recommendations to support these actions are identified for Western, colonial-grounded institutions, functions, and processes related to research, development and implementation of research funding opportunities, and academics and researchers/trainees in general.
